# In Vitro Digestion and Fecal Fermentation of Peach Gum Polysaccharides with Different Molecular Weights and Their Impacts on Gut Microbiota

**DOI:** 10.3390/foods11243970

**Published:** 2022-12-08

**Authors:** Chaoyang Wei, Li Yao, Lin Zhang, Yu Zhang, Qian Luo, Shuyi Qiu, Xiangyong Zeng, Shiguo Chen, Xingqian Ye

**Affiliations:** 1 Key Laboratory of Fermentation Engineering and Biological Pharmacy of Guizhou Province, School of Liquor and Food Engineering, Guizhou University, Guiyang 550025, China; 2 Key Laboratory of Plant Resource Conservation and Germplasm Innovation in Mountainous Region (Ministry of Education), Institute of Agro-Bioengineering, College of Life Sciences, Guizhou University, Guiyang 550025, China; 3 Laboratory of Food Processing and Engineering, Department of Food Science and Nutrition, College of Biosystems Engineering and Food Science, Zhejiang University, Hangzhou 310058, China; 4 School of Health Science and Food Engineering, University of Shanghai for Science and Technology, Shanghai 200093, China; 5 Guiyang Agricultural Experimental Center, Guiyang Agriculture and Rural Bureau, Guiyang 550018, China

**Keywords:** peach gum polysaccharides, in vitro digestion, fecal fermentation, gut microbiota, dominant bacteria

## Abstract

In the present study, we investigated the in vitro digestion and fermentation characteristics of three peach gum polysaccharides (PGPs) of different molecular weights; i.e., AEPG2 (1.64 × 10^7^ g/mol), DPG2 (5.21 × 10^5^ g/mol), and LP100R (8.50 × 10^4^ g/mol). We observed that PGPs were indigestible during the oral, gastrointestinal, and intestinal stages. However, they were utilized by the gut microbiota with utilization rates in the order of DPG2 > AEPG2 > LP100R. Furthermore, arabinose in PGPs was preferentially utilized by the gut microbiota followed by galactose and xylose. Fermentation of peach gum polysaccharides could significantly increase the production of short-chain fatty acids (SCFAs), especially n-butyric acid. In addition, PGPs with different molecular weights values were predominantly fermented by different bacterial species. AEPG2 and DPG2 were fermented by the *Bacteroidetes* bacteria *Bacteroides*, while the dominant *n*-butyrate-producing bacteria was *Faecalibacterium*. While the LP100R was fermented by *Bacteroides*, *Parabacteroides*, *Phascolarctobacterium*, *Dialister*, *Lachnospiraceae*, and *Blautia*, the dominant n-butyrate-producing bacteria was *Megamonas*. These results indicated that PGPs are potential prebiotics for the food industry.

## 1. Introduction

Peach gum is a common traditional Chinese medicine used to treat dysentery and alleviate pain that is recorded in the *Compendium of Materia Medica*. Peach gum polysaccharides (PGPs) are key components of peach gum secreted from the branches of the peach tree. PGPs are arabinogalactans that are mainly composed of arabinose and galactose with smaller amounts of xylose, glucuronic acid, and mannose [1,2]. The main chain of PGPs consists of a mixture of (1→3)- and (1→6)-linked Galp units; the O-3 and O-4 of the Galp unit are the substitution sites of the branched chain [3]. Recent studies suggested that PGPs can be used as adsorbents [4] and in encapsulation technologies [5], pharmaceuticals, and food [6]. Thus, they have attracted attention due to their versatile properties.

Polysaccharides can alter the composition of gut microbiota to promote human health. For instance, polysaccharides from loquat (*Eriobotrya japonica* L.) leaves and *Tremella fuciformis* increased the relative abundance of some potentially beneficial bacteria [7,8]. Research on non-starch polysaccharides (NSPs) derived from plants and their metabolites has been growing rapidly in recent years. NSPs are not digested in the upper gastrointestinal tract but are enzymatically fermented by gut microbiota in the large intestine, which affects the host’s health through their short-chain fatty acid (SCFA) metabolites [9,10]. Therefore, polysaccharides used as prebiotics can improve the treatment outcomes of metabolic diseases by regulating the gut microbiota composition [11], thereby exerting a positive effect on human health.

To our knowledge, there have been no studies on the digestion and fermentation characteristics of PGPs in the gastrointestinal tract and their effects on intestinal health. In this study, we used an in vitro digestion and fermentation model to investigate the changes in PGPs of different molecular weights during digestion and fermentation and their effects on gut microbiota composition. We hypothesized that PGPs could change the gut microbiota composition and that different bacteria would dominate the fermentation of PGPs with different molecular weights. The results of this study will contribute to a deeper understanding of the digestion and fermentation characteristics of PGPs in humans.

## 2. Materials and Methods

### 2.1. Materials

Raw peach gum was collected from the trunk and branches of white peach (*Prunus persica* Batsch) trees in Yuan Dong Township, Jinhua City, Zhejiang Province, China, and dried by sun exposure and prepared by crushing (80 mesh) in an ultra-micro pulverizer. All other chemical reagents and drugs were of analytical grade.

### 2.2. Preparation and Physicochemical Properties of PGPs

The AEPG2 (the high-molecular-weight (Mw) peach gum polysaccharide that was extracted with 2 M NaOH) and LP100R (the low-Mw peach gum polysaccharide that was degraded from AEPG2 and fractionated by using a 100 kDa ultrafiltration membrane) were obtained from our previous work [12,13]. The DPG2 (a medium-molecular-weight peach gum polysaccharide) was obtained via the degradation of AEPG2 using the Fenton-Cu^2+^ system (Figure S1). The determinations of the monosaccharide composition and Mw were performed according to our previously described method [12,13].

### 2.3. Simulated In Vitro Digestion of PGPs

**Figure 1:**
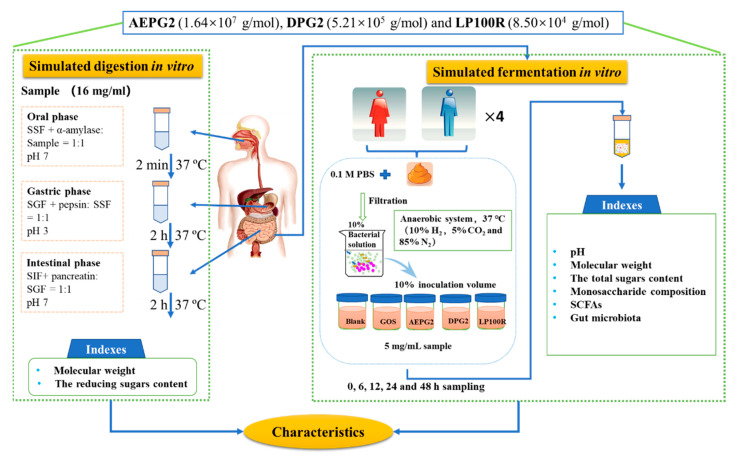
Scheme of in vitro digestion and fermentation of peach gum polysaccharides. AEPG2, alkali (2 M NaOH) extracted PGPs; DPG2, PGPs degraded from AEPG2; LP100R, low-molecular-weight PGPs degraded from AEPG2 and fractionated by using a 100 kDa ultrafiltration membrane; GOS, galactooligosaccharides (positive control); Blank, ddH_2_O (negative control); SSF, salivary-simulating fluid; SGF, gastric-simulating fluid; SIF, small-intestine-simulating fluid. Each experiment had three replications (*n* = 3).

In vitro oral, stomach, and small intestinal digestion was simulated as described previously [14]. The salivary-simulating fluid (SSF), gastric-simulating fluid (SGF), and small-intestine-simulating fluid (SIF) consisted of the corresponding electrolytes, enzymes, and water (Table S1). First, 16 mg/mL of the AEPG2, DPG2, and LP100R solutions were prepared according to the scheme shown in Figure 1. Briefly, 10 mL of the polysaccharide solution was mixed with 10 mL of SSF (7.0 mL SSF stock solution, 1 mL of 1500 U/L α-amylase, 50 µL of 0.3 M CaCl_2_, and 1.95 mL of distilled water), and the pH was adjusted to 7.0 using 1 M NaOH. The digestion solution was placed in a water bath at 37 °C for 0 and 2 min. After simulating salivary digestion, 20 mL of the oral digestive sample was mixed with 20 mL of SGF (15 mL of SGF stock solution, 3.2 mL of porcine pepsin (25,000 U/L), 10 µL of 0.3 M CaCl_2_, and 1.39 µL of distilled water). The pH of the sample was adjusted to 3.0 using 0.1 M HCl, and the sample was placed in a water bath at 37 °C for 0, 0.5, 1.0, 1.5, and 2 h. After simulating gastric digestion, 20 mL of the gastric digestive samples was mixed with 20 mL of SIF (11 mL of SIF stock solution, 5 mL of the pancreatin (800 U/L), 2.5 mL of fresh bile, 40 µL of 0.3 M CaCl_2_, and 1.31 mL of distilled water). The pH of the sample was adjusted to 7.0 using 1 M NaOH, and the sample was incubated in a water bath at 37 °C for 0, 0.5, 1.0, 1.5, and 2 h. Then, 3 mL of the digest was taken from each tube and immediately placed in boiling water for 10 min to destroy the enzymes. The reducing sugar content was determined using the dinitrosalicylic acid (DNS) method [15], and glucose was used as the standard (Table S2). The molecular-weight determination of the PGPs during digestion was performed using SEC-MALLS according to our previously described method [12,13].

### 2.4. In Vitro Simulated Fermentation of PGPs

#### 2.4.1. Preparation of Human Fecal Microbiota

The fermentation in vitro was based on the method by Li et al. [16]. On the same day, the fresh human fecal samples were collected from 8 healthy volunteers (no history of gastrointestinal disease, no antibiotics for 3 months, and a healthy diet) and immediately placed at 37 °C in an anaerobic incubator (10% H_2_, 5% CO_2_, and 85% N_2_) as shown in Figure 1. Equal amounts of feces were mixed and immediately added (*v*/*v*) to a 0.1 M PBS solution (pH 7.4) to form a 10% (*w*/*w*) solid–liquid mixture followed immediately by fully vortex shaking for 1 min and filtering through a double layer of nylon gauze. The filtrate was collected for later use.

#### 2.4.2. PGPs Fermentation In Vitro

The in vitro fermentation of PGPs was performed using a previously described method with slight modifications [17]. The basal nutrient medium contained 2.0 g/L yeast extract, 2.0 g/L peptone, 0.1 g/L NaCl, 0.04 g/L KH_2_PO_4_, 0.04 g/L K_2_HPO_4_, 0.01 g/L MgSO_4_·7HO_2_, 0.01 g/L CaCl_2_, 2 g/L NaHCO_3_, 0.02 g/L hemoglobin chloride, 0.5 g/L cysteine-HCl 0.5 g/L bile salts, 2.0 mL/L Tween80, and 1.0 mL/L 1% resin aspartame at pH 7.4. The AEPG2, DPG2, LP100R, and GOS (galactooligosaccharides, positive control) were dissolved in the medium to form a fermentation broth at a concentration of 5 mg/mL. The Blank was the medium with ddH_2_O (negative control). All of these samples were exposed in an anaerobic incubator (Shanghai Yuejin Medical Instruments, Shanghai, China) before being transferred into pre-sterilized anaerobic tubes.

Next, 1 mL of human fecal microbiota solution at a concentration of 10% (*w*/*w*) was mixed with 9 mL of culture solution containing PGPs in a sealed anaerobic tube and vortexed well in a vortexer as the polysaccharides group. Then, 9 mL of culture solution without PGPs was used instead of the polysaccharide solution (Blank) and the other steps followed as above. All sealed anaerobic tubes were incubated at 37 °C in the anaerobic incubator. The entire process was carried out in an anaerobic system (10% H_2_, 5% CO_2_, and 85% N_2_). The fermentation broth was then removed and stored at −80 °C for further study at five time points: 0, 6, 12, 24, and 48 h.

#### 2.4.3. Determination of Chemical Indices in the Fermentation Broth during Fermentation

The fermentation products from the PGP groups, the GOS, and the Blank were centrifuged at 10,000× *g* for 15 min at the five-time points of 0, 6, 12, 24, and 48 h, and then the supernatant was transferred to a 10 mL capped centrifuge tube. The total sugar content was measured via the phenol-sulfuric acid method [18]; glucose was used as the standard (Table S2). The determinations of the molecular weight and monosaccharide composition of the PGPs during fermentation were performed using SEC-MALLS and pre-column derivatization according to our previously described method [12,13]. The pH of the fermentation products was measured using a pH meter.

#### 2.4.4. Determination of SCFA Content during Fermentation

The supernatant of the fermentation broth was passed through a 0.22 μm filter membrane, and then the composition and content of the SCFAs were determined using gas chromatography [19]. The chromatographic analysis was carried out on an Agilent 6890N gas chromatograph (Agilent Technologies, Santa Clara, CA, USA) and an HP-INNOWAX column (0.32 mm × 30 m, 0.25 μm Agilent Technologies, Santa Clara, CA, USA). The GC conditions were an FID detector with N_2_ carrier gas. The flow rate of N_2_ was 19.0 mL/min, and the split ratio was 1:10. The airflow rate was 300 mL/min, and the H2 flow rate was 30 mL/min. The temperature of the detector and the injector was 240 °C. The heating procedure was conducted from 100 °C (0.5 min) to 180 °C (4 °C/min). The sample injection volume was 1 µL, and the determination time was 20.5 min. Data were analyzed using HP Chem workstation software (A.09, Agilent). The content of SCFAs was calculated with a standard curve as shown in Table S2.

#### 2.4.5. Determination of the Composition of Gut Microbiota

After fermentation for 48 h, the broths of the Blank, GOS, AEPG2, DPG2, and LP100R were centrifuged (8000× *g*, 10 min), and all of the bacterial DNA of each treatment group was extracted separately using a TIANamp Stool DNA kit (Miki Biotechnology Co., Ltd., Guangzhou, China) according to the instructions. An ABI GeneAmp^®^ 9700 PCR thermocycler (ABI, CA, USA) was used to amplify the hypervariable V3-4 region of the bacterial 16S rRNA gene using the primer pairs 338F (5′-ACTCCTACGGGAGGCAGCAG-3′) and 806R (5′-GGACTACHVGGGTWTCTAAT-3′). Purified amplicons were combined in equimolar groups and paired-end sequenced on an Illumina NovaSeq PE250 platform (Illumina, San Diego, CA, USA). The raw 16S rRNA gene sequences were merged using FLASH (v 1.2.8), and sequences with 97% similarity were classified as operational taxonomic units (OTUs). The taxonomic assignment was carried out with Silva (http://www.arb-silva.de, accessed on 10 November 2022.). The alpha values for the Kruskal–Wallis and Wilcoxon tests were set to 0.05, while the threshold of the logarithmic LDA score for discriminative features was less than 3.0.

### 2.5. Statistical Analysis

Each experiment had three replications, and the data were expressed as the mean ± standard deviation (SD). The statistical analysis of the data was performed using SPSS Statistics 17.0 software; an ANOVA one-way analysis followed by a Tukey’s test (*p* < 0.05) were used to evaluate the significance of the differences between the data.

## 3. Results and Discussion

### 3.1. Monosaccharide Composition and Molecular Weight of PGPs

We obtained three PGPs with different molecular weights (Table 1): AEPG2 (1.64 × 10^7^ g/mol), DPG2 (5.21 × 10^5^ g/mol), and LP100R (8.50 × 10^4^ g/mol). AEPG2 was a high-molecular-weight polysaccharide extracted from peach gum using 2 M NaOH. The DPG2 and LP100R had different molecular weights, but both were derived via degradation from AEPG2. The DPG2 was a homogeneous fraction (Figure S1B); its monosaccharide composition was similar to that of the AEPG2 and LP100R prepared in our previous study [12,13]. All of the PGPs were type II arabinogalactans that were mainly composed of arabinose and galactose with similar structures (Figure S1C). Thus, we compared the digestion and fermentation characteristics of the three PGPs.

### 3.2. Characteristics of PGPs during In Vitro Digestion

**Figure 2:**
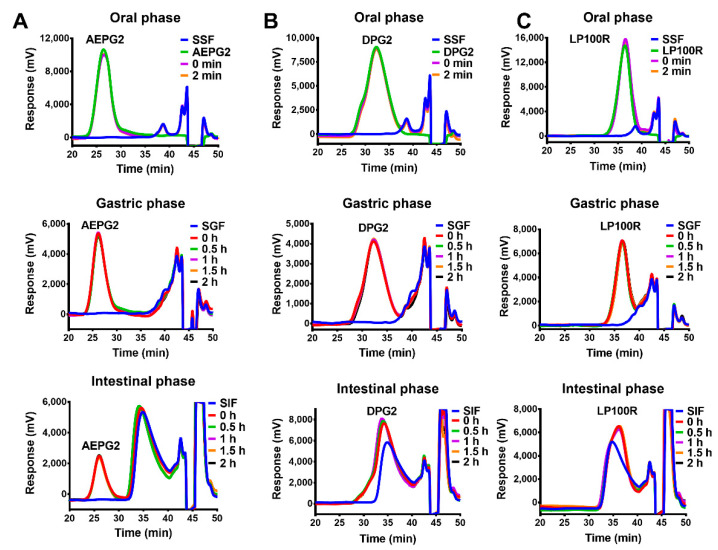
Changes in molecular weights of PGPs in the digestive process: (**A**) AEPG2; (**B**) DPG2; (**C**) LP100R.

As shown in Figure 2, the gel permeation chromatography (GPC) retention times of the AEPG2, DPG2, and LP100R after 0 and 2 min of salivary-stimulation treatment remained unchanged, which indicated a negligible change in the molecular weights. Similar chromatograms were also observed after 0–2 h of treatment in the gastric- and small-intestine-simulation stages. The chromatograms corresponding to the DPG2 and LP100R in the small-intestine-simulation stage differed slightly from their corresponding chromatograms in the salivary and gastric phases. However, no reducing sugar was detected in the digestion solution, possibly due to the interaction between the enzymes (pepsin and pancreatic enzymes) and PGPs. The results showed that, consistent with the previous reports [17,20], the AEPG2, DPG2, and LP100R were not digested during the salivary–gastric–small-intestine phase.

### 3.3. Characteristics of PGPs during In Vitro Fermentation

#### 3.3.1. Carbohydrate Consumption during Fermentation

**Figure 3:**
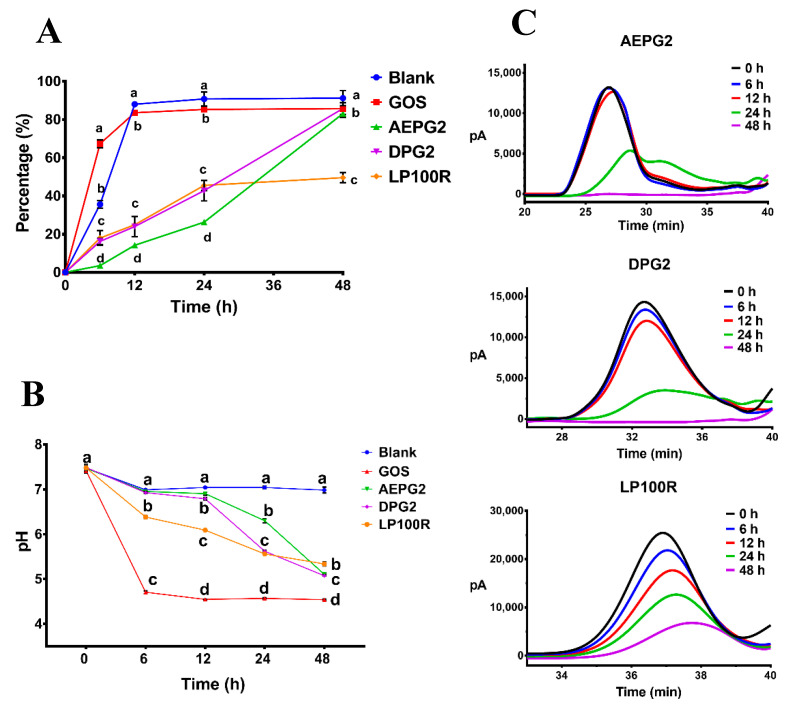
Consumptions of carbohydrates (**A**), changes in pH (**B**), and HPGPC chromatograms of PGPs (**C**) in fermentation broth at different time points of fermentation in vitro. Different lowercase letters indicate significant differences (*p* < 0.05) among different samples at the same time. *n* = 3.

The total sugar contents of the fermentation broths at different fermentation time intervals were measured to evaluate the carbohydrate consumption by the gut microbiota. The total sugar contents of the GOS, AEPG2, DPG2, and LP100R at the end of fermentation were 14.29%, 17.32%, 14.10%, and 50.43% of the initial levels, respectively (Table S3). The results showed that the residual total sugar contents in each treatment group significantly decreased as fermentation time increased, mainly due to their consumption by the gut microbiota. As shown in Figure 3A, the rate of carbohydrate utilization by the gut microbiota followed the order of GOS > DPG2 > AEPG2 > LP100R.

#### 3.3.2. Changes in the pH of the Fermentation Broth

As shown in Figure 3B, the pH of the five groups (7.4 initially) gradually decreased as the fermentation time increased. The pH of the PGP groups was consistently lower than that of the Blank over the tested time range due to a significant increase in the production of SCFAs. The pH trends in the PGP groups within 24 h followed the order of LP100R < DPG2 < AEPG2. At the end of fermentation, the DPG2 and AEPG2, which had higher molecular weights, exhibited greater acidity and pH reduction than the LP100R. This result was consistent with the efficiency of sugar fraction utilization by the gut microbiota for the three PGPs.

#### 3.3.3. Changes in the Molecular Weights of PGPs during Fermentation

As shown in Figure 3C and Table S4, the retention time of PGPs was delayed as the fermentation time increased, which significantly reduced their corresponding peak areas. This indicated that the three PGPs could be degraded and utilized by the gut microbiota, which decreased the molecular weight and content of PGPs. During the 0–12 h fermentation stage, the trend of peak area reduction in the PGPs was LP100R > DPG2 > AEPG2, suggesting that it was easier for the gut microbiota to degrade PGPs with a lower molecular weight. After 24 h of fermentation, the peak areas corresponding to the AEPG2, DPG2, and LP100R were 76.16%, 33.40%, and 56.21% of the initial peak areas, respectively (Table S4). After 48 h of fermentation, the AEPG2 and DPG2 were completely degraded, and their degradation efficiencies were significantly greater than that of the LP100R. Therefore, there were significant differences in the degradation efficiencies of the PGPs with high and low molecular weights by the gut microbiota.

#### 3.3.4. Changes in Monosaccharide Composition of PGPs during Fermentation

**Figure 4:**
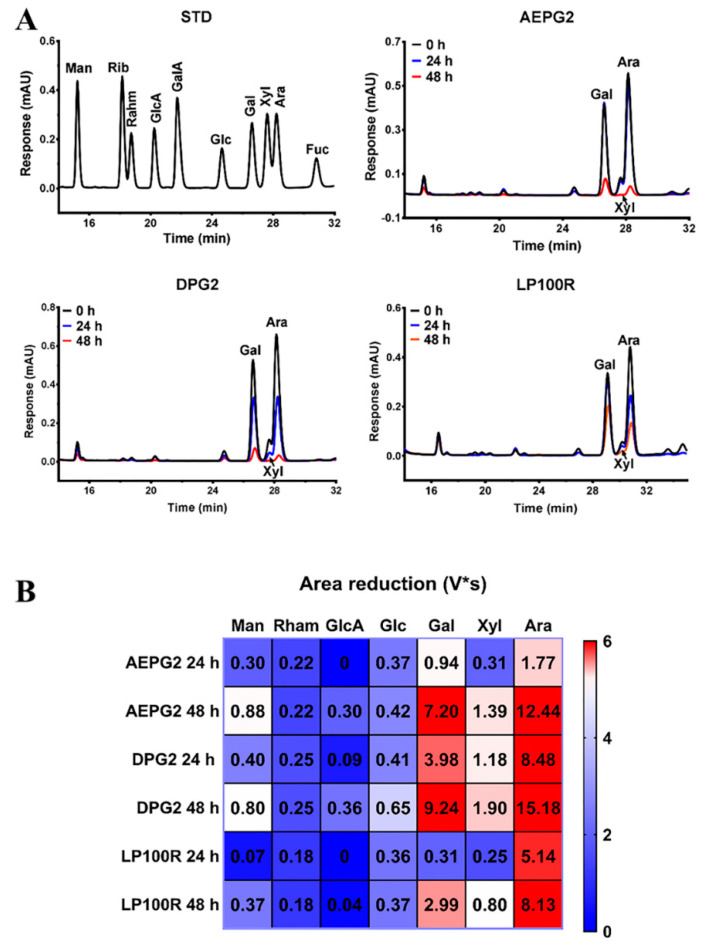
(**A**) Monosaccharide compositions of PGPs at different time points of fermentation in vitro; (**B**) heat map of monosaccharide area reduction of PGPs based on 0 h in vitro fermentation vs. 24 and 48 h. STD, standard monosaccharide mixture.

The above results showed that the PGPs could be degraded and utilized by the gut microbiota with different degradation and utilization patterns for different PGPs. Among the monosaccharides, major content changes were seen for arabinose, galactose, and xylose before and after fermentation (Figure 4A). The in vitro fermentation consumption of the three monosaccharides is shown in Table S5. The consumption of arabinose, galactose, and xylose in both the AEPG2 and DPG2 was >80% after 48 h of fermentation, which far exceeded that of the LP100R. This was consistent with the above conclusion regarding the degradation efficiencies of these three PGPs. In terms of the individual monosaccharide peak areas (Figure 4B), the area reduction of galactose, xylose, and arabinose increased significantly with time, and the area reduction of arabinose was significantly greater than that of galactose, which indicated that the gut microbiota preferentially utilized arabinose followed by galactose and xylose. Our previous work showed that PGPs had β-(1→6)-galactose as the main chain and a high content of arabinose and xylose as side chains [12]. Previous studies confirmed that polysaccharide consumption was closely related to its structure [21,22]. For instance, monosaccharides located in the side chain were more easily degraded by the gut microbiota than those located in the main chain [23]. Therefore, PGPs may be degraded in the same manner.

#### 3.3.5. Changes in SCFA Contents

**Figure 5:**
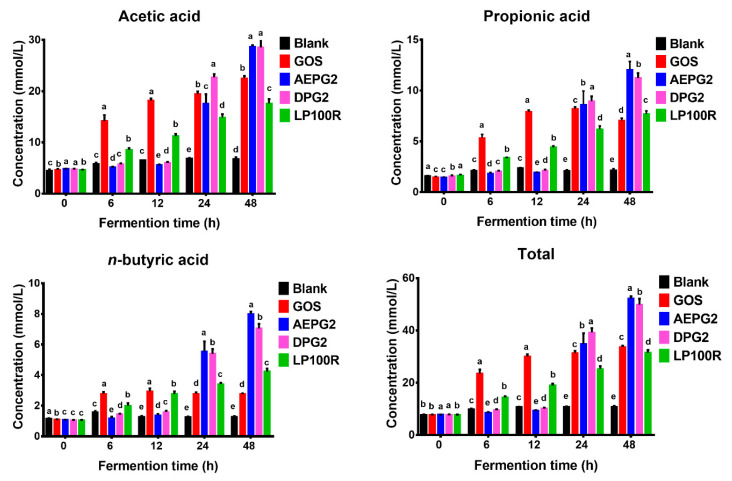
Concentrations of SCFAs in fermentation broths at different time points of fermentation in vitro. Different lowercase letters indicate significant differences (*p* < 0.05) among different samples at the same time. *n* = 3.

The fermentation of the PGPs by the gut microbiota produced significant changes in the SCFA levels (Table S6). The acetic acid, propionic acid, and n-butyric acid contents were significantly higher in the PGP groups (AEPG2, DPG2, and LP100R) than in the Blank group at the end of the fermentation (Figure 5). Reportedly, the fermentation of xylose and glucuronic acid significantly increased the acetic acid and n-butyric acid contents and the fermentations of arabinose, and xylose significantly increased the propionic acid content [24]. As shown in Figure 5, the fermentation of GOS significantly increased the acetic acid and propionic acid contents, suggesting that galactose contributed more to acetic acid and propionic acid than to n-butyric acid. This might explain why the contents of propionic acid and n-butyric acid were significantly higher in the PGP groups than in the GOS group.

Acetic acid, propionic acid, and n-butyric acid are beneficial to human health. Acetic acid can be utilized as an energy source in organ tissues; propionic acid is associated with cholesterol metabolism; and n-butyric acid, as an energy source for intestinal epithelial cells, exhibits immunological, anti-inflammatory, and anti-cancer properties [25]. Our results indicated that the SCFAs of the PGPs fermented by the gut microbiota might benefit host health. The total amount of SCFAs among these five groups was in the order of AEPG2 > DPG2 > GOS > LP100R > Blank, which indicated that the high-molecular-weight PGPs increased the SCFA contents more than the low-molecular-weight PGPs.

### 3.4. Effect of PGPs on Microbial Communities

**Figure 6:**
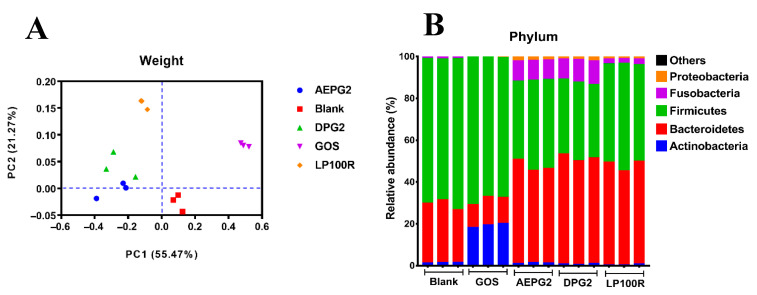
(**A**) Principal coordinates analysis of gut microbiota; (**B**) gut microbial composition at phylum level.

We pre-clustered the obtained unique tag sequences to effectively reduce the number of incorrect OTUs. Then, UCLUST was used to calculate the OTUs of the pre-clustered tags at a 0.03 distance (i.e., 97% similarity). The numbers of OTUs and valid tags for each sample are listed in Table 2. The tag with the highest number of OTUs was selected as the OTU representative sequence and compared with the Greengenes database to obtain the corresponding alignment sequence. The annotation results were analyzed statistically. There are various measures of alpha diversity; in this experiment, Chao1, Shannon, Simpson, OTU, and Goods coverage were used to evaluate the community diversity (Figure S2). We observed that the intestinal microbial community could reflect the diversity of the samples and the richness of the biocoenosis (Table 2). A principal coordinate analysis (PCoA) was used to identify the overall differences in the gut microbiota of the different treatment groups. As shown in Figure 6A, the cumulative variance contribution of the two principal component factors PC1 and PC2 was 76.74%, indicating that most of the information of the different treatment groups could be explained. The more similar the composition of the gut microbiota, the closer they were to the PCoA plot. The PGP groups, GOS, and Blank were distinctly separated from each other in the PCoA plot, which indicated significant differences in the gut microbiota composition among these groups. In the PGP groups, the AEPG2 and DPG2 were close to each other and distant from the LP100R in the PCoA plots, which indicated significant differences in the compositions of the gut microbiota between PGPs with high and low molecular weights.

The GOS and PGPs had significantly different effects on the gut microbiota composition at the phylum level after 48 h of fermentation. As shown in Figure 6B, the relative abundance of *Actinobacteria*, *Bacteroidetes*, and *Firmicutes* in the Blank were 1.56%, 27.91%, and 69.47%, respectively. Compared with the Blank, the relative abundances of *Actinobacteria* (19.32%) and *Firmicutes* (71.08%) in the GOS group and that of *Bacteroidetes* in the PGP groups (AEPG2, DPG2, and LP100R) were increased (by 46.36%, 50.89%, and 47.74%, respectively). *Bacteroidetes*—members of the polysaccharide degradation consortium—produce energy from dietary fiber and starch and might be a major source of propionate [26]. The composition of the gut microbiota also changed significantly at the class, order and family levels based on the type of treatment (Figure S3).

**Figure 7:**
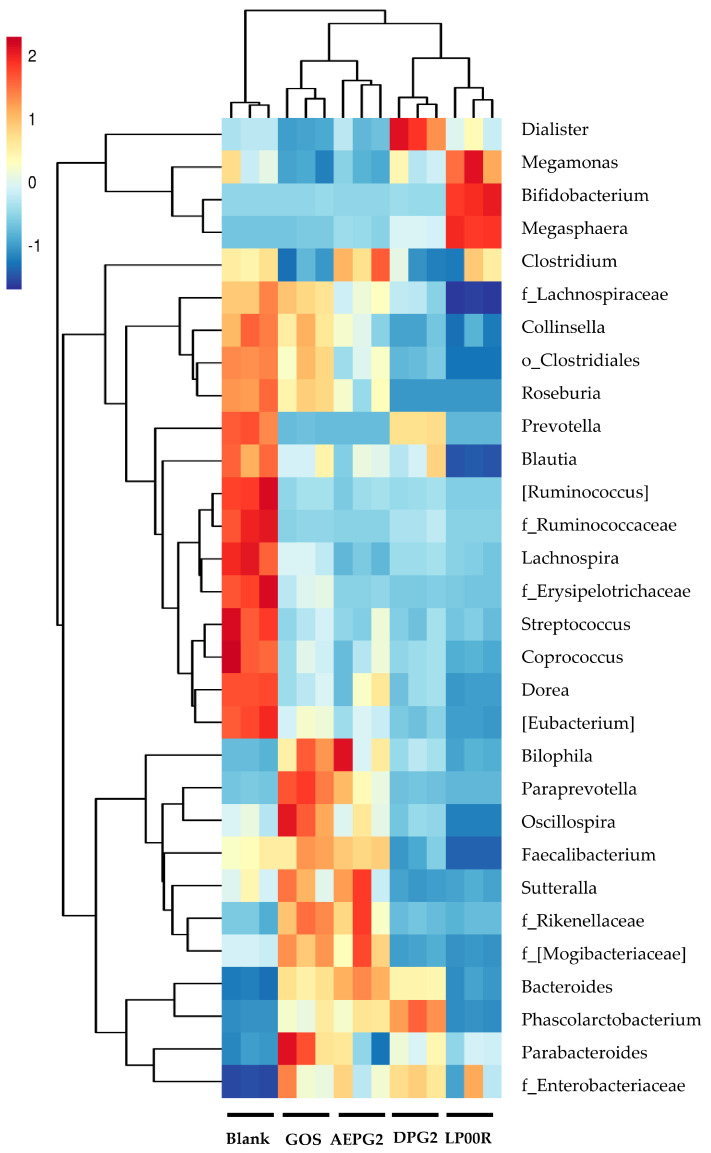
Heat map of gut microbial composition at the genus level.

The linear discriminant analysis (LDA) effect size (LEFSe) results identified 37 taxa at different levels (LDA > 4) and showed different bacterial taxa abundances in the Blank (11), GOS (8), AEPG2 (9), DPG2 (7), and LP100R (1) (Figure S4). At the genus level, the dominant microbes in the Blank, GOS, AEPG2, DPG2, and LP100R were *Prevotella*, *Faecalibacterium*, *Blautia*, *Pseudobutyrivibrio*, and *Dorea*; *Megasphaera*, *Bifidobacterium*, and *Megamonas*; *Dehalobacterium* and *Paraprevotella*; *Bacteroides* and *Fusobacterium*; and *Dialister*, respectively. We compared the relative abundances of the top 30 genera and found that *Bifidobacterium*, *Bacteroides*, *Parabacteroides*, *Blautia*, *Faecalibacterium*, *Megamonas*, and *Phascolarctobacterium* were dominant over all the intestinal core bacteria genera (Figure 7). The relative abundances of *Bifidobacterium* and *Megamonas* were significantly higher in the GOS group than in the Blank group, indicating that these bacteria were involved in the fermentation of GOSs. Similarly, *Bacteroides*, *Parabacteroides*, *Dialister*, *Faecalibacterium*, and *Phascolarctobacterium* were involved in the fermentation and utilization of PGPs. Different bacteria dominated the fermentation of PGPs with different molecular weights (Table 3). For instance, the relative abundance of *Bacteroides*, which belongs to *Bacteroidetes*, in the AEPG2 and DPG2 groups was significantly greater than that in the LP100R group. The AEPG2 and DPG2 significantly increased the relative abundance of *Faecalibacterium*, while the LP100R significantly increased the relative abundance of *Megamonas*, which indicated differences in n-butyric acid production between the LP100R and AEPG2 and DPG2. Similarly, the relative abundances of *Dialister* and *Phascolarctobacterium* in the LP100R group were higher than those in the AEPG2 and DPG2 groups.

The main bacterial genus involved in the fermentation of PGPs was *Bacteroides* (the relative abundances were 37.82%, 45.83%, and 16.92% for the AEPG2, DPG2, and LP100R groups, respectively), which also ferments and metabolizes most dietary fiber in foods to produce SCFAs [7,27]. Our study also found similar results for SCFA production (Figure 5). The relative abundances of *Parabacteroides*, *Faecalibacterium*, *Dialister*, and *Phascolarctobacterium* were higher in the PGP groups than in the GOS and Blank groups. *Parabacteroides* cause metabolic dysfunction by producing secondary bile acids and succinic acids, which are negatively correlated with obesity [28]. *Faecalibacterium* has been reported to produce butyric acid, which is involved in intestinal inflammation and sleep regulation [29,30]. *Dialister* can decarboxylate succinate to propionate [31]. Previous studies have found that propionic acid acts as an important mediator between nutrition, gut microbiota, and physiology. It also reduces fatty acid content in the liver and plasma, reduces food intake, exerts immunosuppressive effects, and may improve tissue insulin sensitivity [32]. *Phascolarctobacterium succinatutens* synthesizes propionate from sugars [33] and protects the colonic mucosa, reduces the effects of colitis, and increases the risk of colon cancer.

In addition, *Lachnospiraceae*, *Blautia*, and *Megamonas* were relatively more abundant in the PGP groups than the GOS and Blank groups. The *Lachnospiraceae* family or specific taxa of *Lachnospiraceae* are involved in inflammatory diseases [34]. *Lachnospiraceae* is an important butyrate producer that resides in the gut microbiome [35] and possibly regulates inflammatory diseases such as metabolic syndrome, diabetes, liver disease, IBD, and CKD. In addition, as a genus in the family *Lachnospiracea*, *Blautia* alleviates inflammatory and metabolic diseases and has antimicrobial activity against specific microorganisms [36]. *Megasphaera* normalizes the production of hyperlactate, thus promoting the production of butyrate [37]. These results suggested that *Bacteroides*, *Parabacteroides*, *Phascolarctobacterium*, *Dialister*, *Faecalibacterium*, *Lachnospiraceae*, *Blautia*, and *Megamonas* are beneficial bacteria for host health and that PGPs can act as novel prebiotics by promoting the growth of these bacteria.

## 4. Conclusions

In this study, we investigated whether PGPs with different molecular weights could be digested and fermented in vitro and studied their differential effects on the gut microbiota. Overall, our results demonstrated that PGPs were not digested in the human upper gastrointestinal tract. However, PGPs were fermented by the gut microbiota in the following order: DPG2 > AEPG2 > LP100R. Furthermore, arabinose in PGPs was preferentially utilized by the gut microbiota followed by galactose and xylose. During in vitro fermentation, the molecular weight of the PGPs and the carbohydrate content significantly decreased, whereas the production of SCFAs significantly increased, especially in the high-molecular-weight PGP groups. Moreover, the PGPs with different molecular weights led to the involvement of different dominant bacteria during fermentation. The gut microbiota structural analysis showed that the PGPs did not change the microbial diversity; however, they changed the gut microbiota composition at different levels. In addition, the fermentation of the PGPs significantly promoted the growth of some bacteria such as *Bacteroides*, *Parabacteroides*, *Phascolarctobacterium*, *Dialister*, *Faecalibacterium*, *Lachnospiraceae*, *Blautia*, and *Megamonas*, which indicated that the intake of PGPs might be beneficial to human intestinal health. Therefore, the present study provided a reference for the function and potential application of PGPs in maintaining intestinal health.

## Figures and Tables

**Table 1:** Monosaccharide compositions and molecular weights of PGPs.

Sample	Monosaccharide Composition (mol %)							Mw (g/mol)
	Mannose	Rhamnose	Glucuronic Acid	Glucose	Galactose	Xylose	Arabinose	
AEPG2	3.08 ± 0.06 ^c^	0.73 ± 0.03 ^b^	3.08 ± 0.06 ^a^	1.54 ± 0.10 ^a^	34.95 ± 0.80 ^c^	7.20 ± 0.10 ^a^	49.41 ± 1.50 ^a^	(1.64 ± 0.04) × 10^7 a^
DPG2	4.64 ± 0.09 ^b^	1.02 ± 0.06 ^a^	2.61 ± 0.09 ^b^	trace	39.82 ± 0.51 ^a^	3.89 ± 0.06 ^b^	48.02 ± 1.4 ^b^	(5.21 ± 0.41) × 10^5 b^
LP100R	5.42 ± 0.11 ^a^	1.00 ± 0.05 ^a^	2.70 ± 0.09 ^b^	trace	35.80 ± 0.43 ^b^	7.04 ± 0.25 ^a^	48.21 ± 1.25 ^b^	(8.5 ± 0.6) × 10^4 c^

Mw, molecular weight; AEPG2, alkali (2 M NaOH) extracted PGPs; DPG2, PGPs degraded from AEPG2; LP100R, low-molecular-weight PGPs degraded from AEPG2 and fractionated by using a 100 kDa ultrafiltration membrane. Different lowercase letters within rows indicate differences (*p* < 0.05) among samples. *n* = 3. Trace: <0.1%.

**Table 2:** Alpha diversity of samples among different treatment groups.

Groups		Indices				
	Total OTUs	Total Tags	Goods Coverage	Chao1	Shannon	Simpson
Blank	1105 ± 24.02 ^a^	74,878 ± 2335 ^e^	1.00 ± 0.00 ^a^	1192.94 ± 57.68 ^ab^	5.73 ± 0.03 ^a^	0.95 ± 0.01 ^ab^
GOS	400 ± 65.83 ^e^	77,712 ± 2411 ^d^	1.00 ± 0.00 ^a^	598.28 ±36.81 ^d^	3.84 ± 0.13 ^b^	0.87 ± 0.01 ^b^
AEPG2	913 ± 75.48 ^b^	91,668 ± 4132 ^b^	1.00 ± 0.00 ^a^	1295.39 ± 94.02 ^a^	5.91 ± 0.26 ^a^	0.96 ± 0.01 ^a^
DPG2	801 ± 14.64 ^d^	81,671 ± 2006 ^c^	1.00 ± 0.00 ^a^	1101.75 ± 99.58b ^c^	5.58 ± 0.26 ^a^	0.95 ± 0.01 ^a^
LP100R	853 ± 87.61 ^c^	120,167 ± 41,739 ^a^	1.00 ± 0.00 ^a^	1028.69 ± 54.38 ^c^	5.59 ± 0.12 ^a^	0.96 ± 0.01 ^a^

Different lowercase letters indicate significant differences (*p* < 0.05) among different groups. *n* = 3.

**Table 3:** Relative abundances of 9 microbes in different groups at the genus level.

Phylum	Family	Genus	Relative Abundances (%)				
			Blank	GOS	AEPG2	DPG2	LP100R
*Actinobacteria*	*Bifidobacteriaceae*	*Bifidobacterium*	-	18.94 ± 0.94 ^a^	0.01 ± 0.00 ^b^	-	-
*Bacteroidetes*	*Bacteroidaceae*	*Bacteroides*	7.19 ± 0.28 ^e^	10.70 ± 0.40 ^d^	37.82 ± 0.89 ^b^	45.83 ± 1.82 ^a^	34.33 ± 0.64 ^c^
*Bacteroidetes*	*Porphyromonadaceae*	*Parabacteroides*	0.67 ± 0.02 ^d^	1.31 ± 0.04 ^c^	2.71 ± 0.16 ^a^	1.21 ± 0.05 ^cd^	1.64 ± 0.07 ^b^
*Firmicutes*	*Lachnospiraceae*	*Blautia*	4.35 ± 0.15 ^a^	0.26 ± 0.01 ^d^	2.48 ± 0.12 ^b^	2.13 ± 0.10 ^c^	2.55 ± 0.12 ^b^
*Firmicutes*	*Lachnospiraceae*		9.23 ± 0.42 ^a^	0.67 ± 0.02 ^e^	8.34 ± 0.40 ^b^	6.15 ± 0.29 ^c^	4.74 ± 0.23 ^d^
*Firmicutes*	*Ruminococcaceae*	*Faecalibacterium*	5.96 ± 0.21 ^c^	0.26 ± 0.01 ^e^	7.93 ± 0.36 ^a^	7.49 ± 0.31 ^b^	2.03 ± 0.11 ^d^
*Firmicutes*	*Veillonellaceae*	*Dialister*	1.12 ± 0.05 ^c^	1.48 ± 0.06 ^b^	0.47 ± 0.01 ^e^	0.83 ± 0.02 ^d^	3.20 ± 0.11 ^a^
*Firmicutes*	*Veillonellaceae*	*Megamonas*	13.04 ± 0.67 ^b^	19.31 ± 0.93 ^a^	7.48 ± 0.36 ^e^	8.62 ± 0.40 ^d^	12.00 ± 0.49 ^c^
*Firmicutes*	*Veillonellaceae*	*Phascolarctobacterium*	1.03 ± 0.03 ^c^	0.97 ± 0.03 ^c^	4.71 ± 0.18 ^bc^	5.28 ± 0.16 ^b^	7.58 ± 0.35 ^a^

Different lowercase letters indicate significant differences (*p* < 0.05) among different groups. *n* = 3.

## Data Availability

The data are contained within the article.

## Supplementary Materials

The following supporting information can be downloaded at: https://www.mdpi.com/article/10.3390/foods11243970/s1, Figure S1: (A) Elution curves of DPG from peach gum on Q-Sepharose Fast Flow column, (B) The chromatography profile of DPG2 fraction on SB-806 and SB-804 HR column. (C) ^1^H NMR spectra of AEPG2, DPG2 and LP100R; Figure S2: Alpha diversity analysis of gut microbiota. Tags length distribution map (A), Rarefaction Curve (B), Shannon index curve (C), Good coverage curve (D), Simpson index curve (E), Rank abundance curve (F); Figure S3: Gut microbial composition at (A) Class level, (B) Order level and (C) Family level; Figure S4: LEfse and LDA analysis of microbiota; Table S1: Preparation of stock solutions of simulated digestion fluids; Table S2: Standard curves; Table S3: Contents of total sugars in fermentation solutions at different time points of fermentation in vitro; Table S4: Distributions of PGPs in fermentation broth at different time points of fermentation in vitro; Table S5: Monosaccharides peak areas of PGPs at different time points of fermentation in vitro;. Table S6: Concentrations of SCFAs in fermentation broths at different time points of fermentation in vitro.
