# Supplementary material for: In Vitro Digestion and Fecal Fermentation of Peach Gum Polysaccharides with Different Molecular Weights and Their Impacts on Gut Microbiota
Source: Foods. 2022 Dec 8;11(24):3970. doi: 10.3390/foods11243970 (PMC9777905; doi:10.3390/foods11243970)
Supplement: Supplementary file 1 [file foods-11-03970-s001.zip › foods-2059592-supplementary.pdf]

Figure supplementary S1

1. Preparation of DPG2

The Fenton-Cu^{2+} system was used to degrade peach gum to prepare medium-molecular peach gum polysaccharides. A suitable amount of AEPG2 was weighed and dissolved in distilled water to form a 0.5% (w/v) solution, 0.5 mmol/L $Cu(Ac)_2$ was added, the pH was adjusted to 6.0, and 0.5% H_2O_2 was added at 25 ºC for 30 min, then 0.6% $NaHSO_3$ was added immediately to terminate the reaction. Then the reaction solution was dialyzed (MWCO 8-14 kDa), concentrated, alcoholic precipitated and lyophilized to obtain degraded peach gum polysaccharides named DPG (73.61%). A QFF ion exchange column was used to elute 10 mg/mL of DPG with a gradient of 0, 0.1, 0.2, 0.3, 0.5 and 2.0 M NaCl at a flow rate of 1.0 mL/min, and an automatic collector was used to collect 6 mL per tube. The distribution of the polysaccharide fractions was detected by the phenol-sulfuric acid method.

2. Isolation of peach gum polysaccharides

As shown in Figure supplementary S1A, A QFF ion exchange column was used to separate two fractions, DPG2 and DPG3, with yields of 80.24% and 19.76%, respectively. The highest yielding fraction, DPG2, was used for purity determination and only one symmetric peak was found in the chromatogram (Figure supplementary S1B), indicating that DPG2 was a homogeneous fraction.

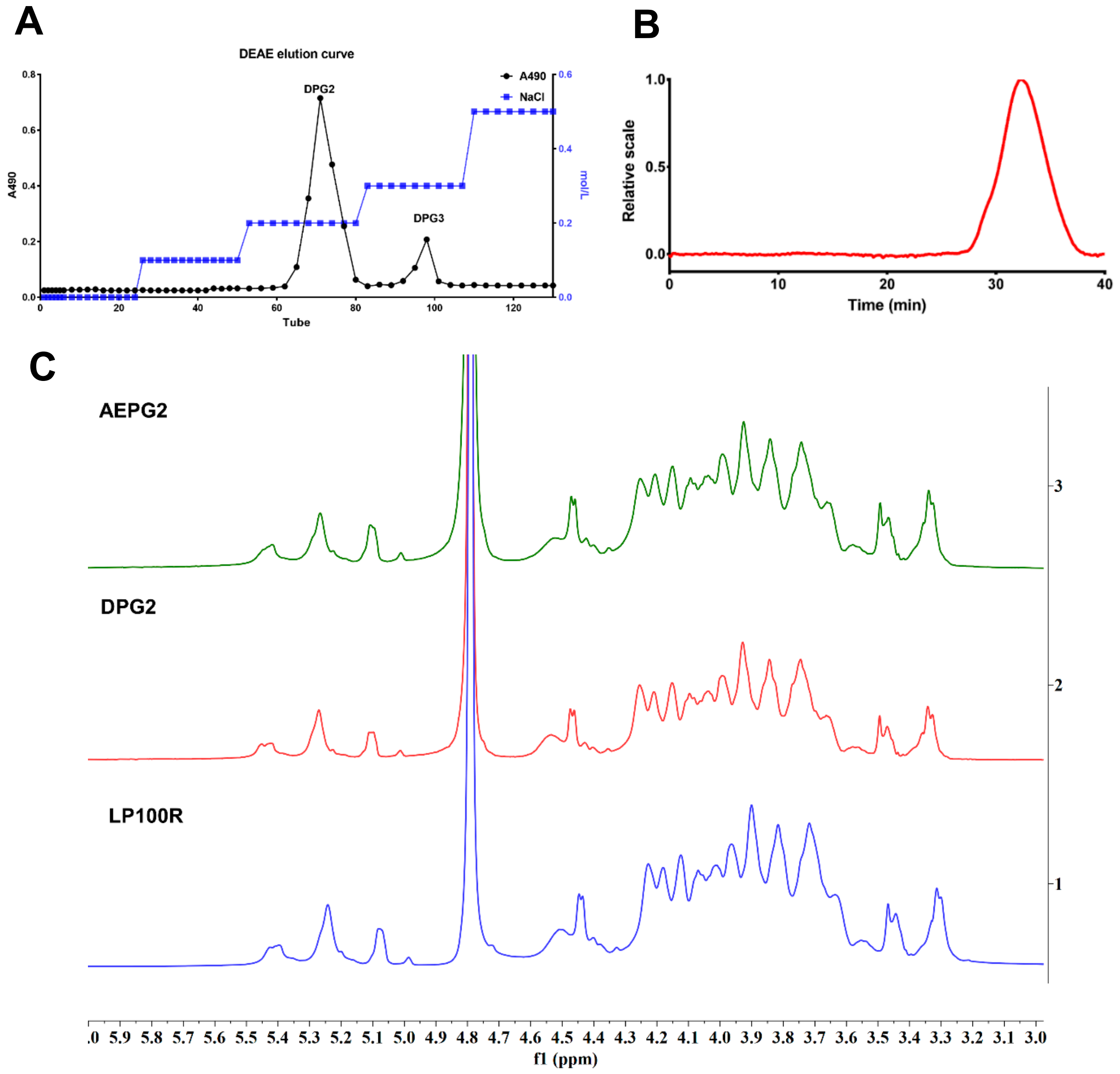

Figure supplementary S1. (A) Elution curves of DPG from peach gum on Q-Sepharose Fast Flow column, (B) The chromatography profile of DPG2 fraction on SB-806 and SB-804 HR column. (C) ^{1}H NMR spectra of AEPG2, DPG2 and LP100R. AEPG2, alkali (2 M NaOH) extracted PGPs; DPG2, degraded PGPs from AEPG2; LP100R, low molecular weight PGPs degraded from AEPG2, and fractionated by using 100 kDa ultrafiltration membrane.

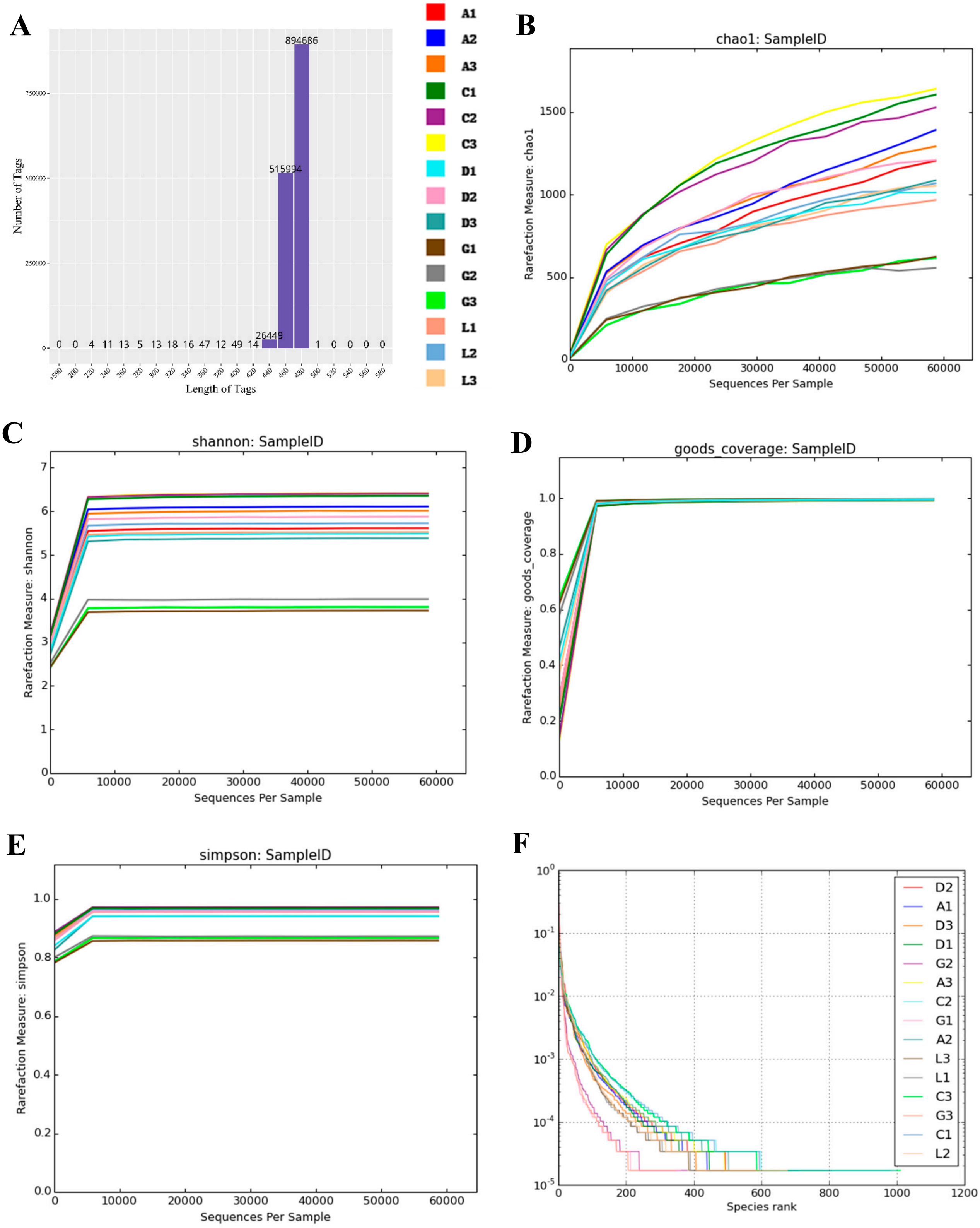

Figure supplementary S2. Alpha diversity analysis of gut microbiota. Tags length distribution map (A), Rarefaction Curve (B), Shannon index curve (C), Good coverage curve (D), Simpson index curve (E), Rank abundance curve (F). A: AEPG2, C: Blank, D: DPG2, G: GOS, L: LP100R.

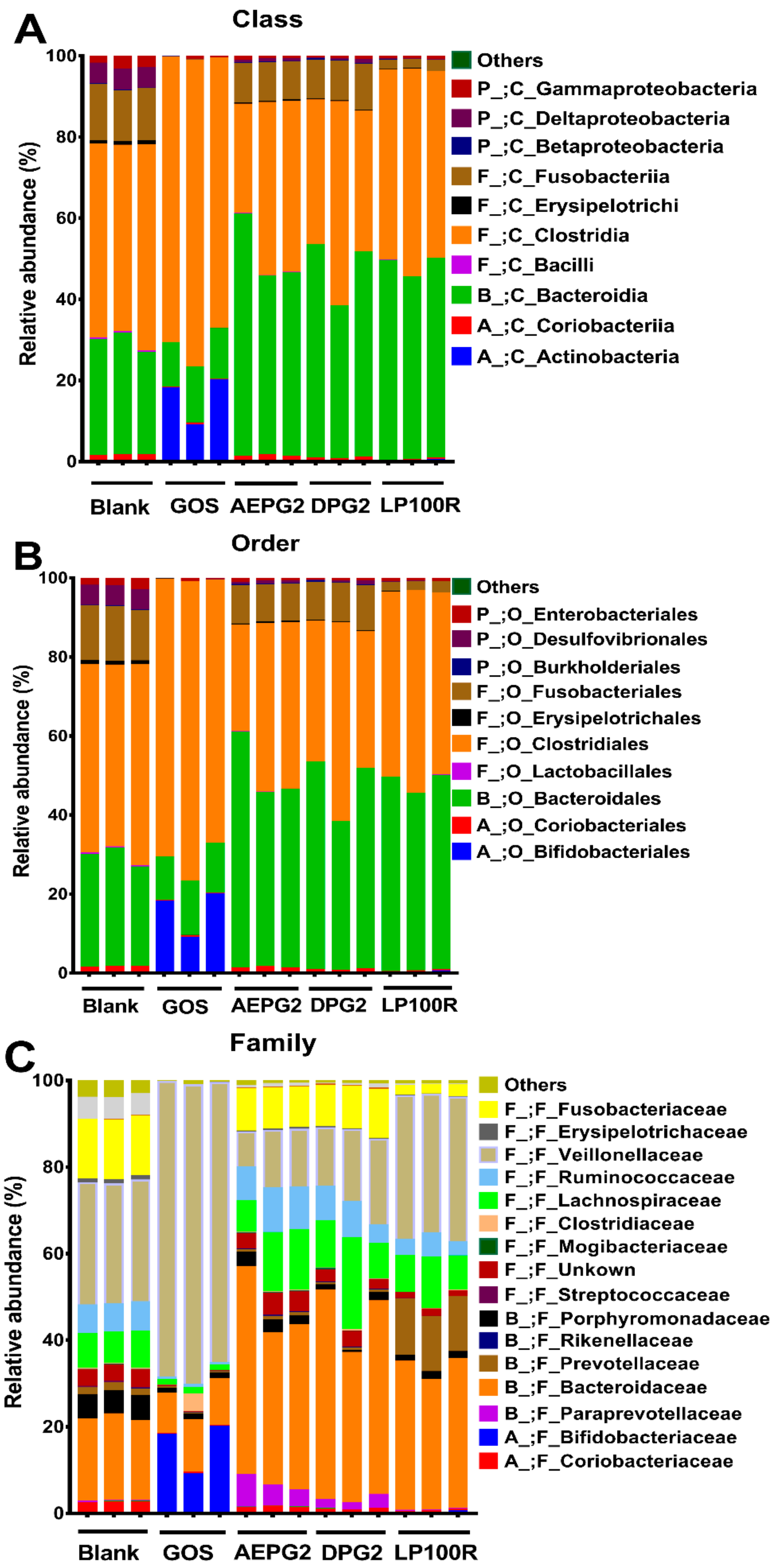

Figure supplementary S3. Gut microbial composition at (A) Class level, (B) Order level and (C) Family level.

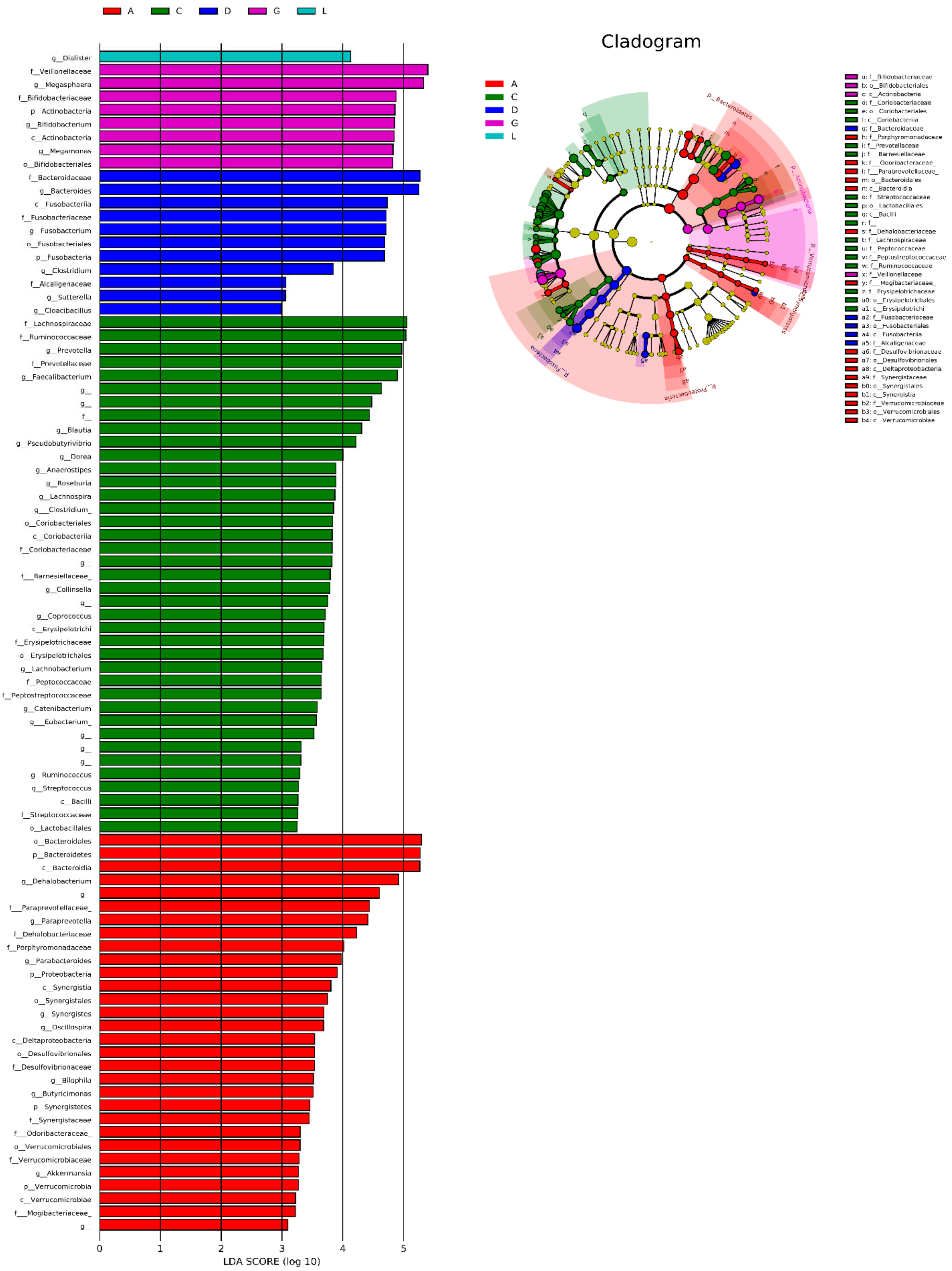

Figure supplementary S4. LEfse and LDA analysis of microbiota. A: AEPG2, C: Blank, D: DPG2, G: GOS, L: LP100R.

Table supplementary S1. Preparation of stock solutions of simulated digestion fluids.

Constituent	Stock concentration		SSF pH 7		SGF pH 3		SIF pH 7	
			Vol. of stock	SSF concentration	Vol. of stock	SGF concentration	Vol. of stock	SIF concentration
	g/L	mol/L	mL	mmol/L	mL	mmol/L	mL	mmol/L
KCl	37.3	0.5	15.1	15.1	6.9	6.9	6.8	6.8
KH_2PO_4	68	0.5	3.7	3.7	0.9	0.9	0.8	0.8
$NaHCO_3$	84	1	6.8	13.6	12.5	25	42.5	85
NaCl	117	2	-	-	11.8	47.2	9.6	38.4
$MgCl_2(H_2O)_6$	30.5	0.15	0.5	0.15	0.4	0.1	1.1	0.33
$(NH_4)_2CO_3$	48	0.5	0.06	0.06	0.5	0.5	-	-
pH adjustment								
	mol/L		mL	mmol/L	mL	mmol/L	mL	mmol/L
NaOH	1		-	-	-	-	-	-
HCl	6		0.09	1.1	1.3	15.6	0.7	8.4

SSF, salivary simulating fluid; SGF, gastric simulating fluid; SIF, small intestine simulating fluid.

Table supplementary S2. Standard curves.

Analytes	Retention time (min)	Standard curves	Correlation coefficient R^2
Total sugars	-	Y = 0.005396X+0.07689	0.999
Reducing sugars	-	Y = 0.374 X+0.04566	0.999
Acetic acid	6.701±0.11	Y=18.864x-10.433	0.999
Propionic acid	8.276±0.21	Y=38.068x-15.690	0.999
Isobutyric acid	8.835±0.15	Y=65.690x-2.330	0.999
Butyric acid	10.127±0.29	Y=51.019x-19.798	0.999
Isovaleric acid	11.021±0.21	Y=91.974x-2.899	0.999
Valeric acid	12.589±0.30	Y=65.445x-3.650	0.999

Table supplementary S3. Contents of total sugars in fermentation solutions at different time points of fermentation *in vitro*

Fermentation time (h)	Total sugars (mg/mL)				
	Blank	GOS	AEPG2	DPG2	LP100R
0	0.36±0.09aB	3.69±0.33aA	3.68±0.40aA	3.67±0.31aA	3.68±0.43aA
6	0.26±0.06bE	1.29±0.24bD	3.36±0.31bA	3.19±0.22bB	3.04±0.34bC
12	0.23±0.02cE	0.69±0.09cD	3.08±0.31cA	2.61±0.21cC	2.78±0.22cB
24	0.23±0.01cE	0.63±0.04dD	2.69±0.25dA	2.13±0.13dB	2.04±0.20dC
48	0.19±0.01dD	0.61±0.03dB	0.69±0.08eB	0.59±0.08eC	1.88±0.15eA

AEPG2, alkali (2 M NaOH) extracted PGPs; DPG2, degraded PGPs from AEPG2; LP100R, Low molecular weight PGPs degraded from AEPG2, and fractionated by using 100 kDa ultrafiltration membrane; GOS, galactooligosaccharides, positive control; Blank, ddH_2O, negative control.

Different lowercase letters indicate significant differences (p < 0.05) in the same group among different time points. Different capital letters indicate significant differences (p < 0.05) among different groups. n=3.

Table supplementary S4. Distributions of PGPs in fermentation broth at different time points of fermentation *in vitro.*

Samples	Culture	Retention time (min)	Area (μv*sec)	Area percentage (%)
AEPG2	0 h	26.76±0.46^{b}	3420919±50636^{a}	100.00±1.33^{a}
	6 h	26.72±0.39^{b}	3395489±99325^{a}	99.26±2.90^{a}
	12 h	27.00±0.23^{b}	3092137±64887^{b}	90.39±1.82^{b}
	24 h	28.65±0.04^{a}	2605308±48324^{c}	76.16±1.41^{c}
	48 h	ND	0±0.0^{d}	0±0.0^{d}
DPG2	0 h	32.68±0.02^{b}	3827056±64205^{a}	100.00±1.68^{a}
	6 h	32.72±0.04^{b}	3436433±42564^{b}	89.79±1.11^{b}
	12 h	32.80±0.03^{b}	2925753±33815^{c}	76.45±0.88^{c}
	24 h	34.39±0.67^{a}	1278411±60682^{d}	33.40±1.79^{d}
	48 h	ND	0±0.0^{e}	0±0.0^{e}
LP100R	0 h	36.85±0.07^{b}	3581444±51915^{a}	100.00±1.45^{a}
	6 h	37.02±0.02^{a}	3144460±48788^{b}	87.80±1.36^{b}
	12 h	37.18±0.01^{a}	2663212±66329^{c}	74.36±1.85^{c}
	24 h	37.30±0.01^{a}	2013305±14736^{d}	56.21±0.41^{d}
	48 h	37.74±0.01^{a}	1215740±96691^{e}	33.95±2.70^{e}

ND is not detected. Different lowercase letters indicate significant differences ($p < 0.05$) in the same column among different time points. n=3.

Table supplementary S5. Monosaccharides peak areas of PGPs at different time points of fermentation *in vitro*.

Samples	Culture	AEPG2		DPG2		LP100R	
Monosaccharide		Area (μv*sec)	percentage（%）	Area (μv*sec)	percentage（%）	Area (μv*sec)	percentage（%）
Gal	0 h	8847944±64553[a]	100.00±1.94[a]	10551747±254563[a]	100.00±7.64[a]	8531720±52248[a]	100.00±4.57[a]
	24 h	8754169±96542[a]	98.94±2.90[a]	6570715±94553[b]	62.27±2.84[b]	8226495±40652[b]	96.42±3.21[b]
	48 h	1652167±64223[b]	18.67±1.93[b]	1307335±65952[c]	12.39±1.98[c]	5541767±35614[c]	64.95±2.57[c]
Xyl	0 h	1450918±15001[a]	100.00±2.85[a]	1954923±135422[a]	100.00±4.06[a]	1187752±39635[a]	100.00±2.99[a]
	24 h	1142279±17965[b]	78.73±2.94[b]	777511±54663[b]	39.77±1.64[b]	940190±5928[b]	79.16±1.98[b]
	48 h	56436±846[c]	3.89±0.36[c]	54927±10667[c]	2.81±0.32[c]	385970±4981[c]	32.50±1.65[c]
Ara	0 h	13545919±151861[a]	100.00±4.56[a]	15775482±148452[a]	100.00±4.45[a]	12056894±245378[a]	100.00±7.36[a]
	24 h	11774266±136453[b]	86.92±4.09[b]	7300393±40535[b]	46.28±3.14[b]	6917468±87443[b]	57.37±2.62[b]
	48 h	1109256±85562[c]	8.19±2.57[c]	594619±4391[c]	3.77±1.33[c]	3923798±53417[c]	32.54±1.64[c]

Different lowercase letters indicate significant differences ($p < 0.05$) in the same group among different time points. n=3.

Table supplementary S6. Concentrations of SCFAs in fermentation broths at different time points of fermentation *in vitro.*

SCFAs (mM)	Sample	Anaerobic fermentation time (h)				
		0	6	12	24	48
Acetic acid	Blank	4.51±0.21^{bD}	5.80±0.24^{cC}	6.52±0.03^{cB}	6.83±0.15^{eA}	6.80±0.30^{dA}
	GOS	4.65±0.20^{bD}	14.16±1.18^{aC}	18.16±0.44^{aB}	19.42±0.51^{bB}	22.50±0.50^{bA}
	AEPG2	4.84±0.04^{aE}	5.15±0.10^{dD}	5.63±0.07^{eC}	17.59±1.81^{cB}	28.69±0.30^{aA}
	DPG2	4.78±0.10^{aE}	5.73±0.19^{cD}	6.05±0.15^{dC}	22.71±0.63^{aB}	28.55±1.47^{aA}
	LP100R	4.61±0.08^{bE}	8.56±0.32^{bD}	11.20±0.45^{bC}	14.86±0.65^{dB}	17.61±0.85^{cA}
Propionic acid	Blank	1.58±0.05^{aC}	2.17±0.07^{cB}	2.37±0.04^{cA}	2.09±0.10^{eB}	2.15±0.16^{eB}
	GOS	1.47±0.07^{cE}	5.34±0.33^{aD}	7.91±0.17^{aB}	8.20±0.21^{cA}	7.04±0.23^{dC}
	AEPG2	1.43±0.05^{cD}	1.81±0.11^{dC}	1.92±0.04^{eC}	8.60±1.35^{bB}	12.05±0.81^{aA}
	DPG2	1.54±0.09^{bD}	2.03±0.07^{cC}	2.11±0.10^{dC}	8.94±0.49^{aB}	11.24±0.48^{bA}
	LP100R	1.62±0.12^{aE}	3.37±0.06^{bD}	4.41±0.15^{bC}	6.18±0.34^{dB}	7.67±0.32^{cA}
i-Butyric acid	Blank	0.10±0.01^{aB}	0.08±0.01^{bB}	0.09±0.01^{cB}	0.11±0.01^{dB}	0.15±0.01^{dA}
	GOS	0.10±0.01^{aD}	0.13±0.01^{aC}	0.21±0.02^{aA}	0.16±0.01^{cB}	0.13±0.01^{dC}
	AEPG2	0.10±0.01^{aC}	0.08±0.01^{bC}	0.09±0.01^{cC}	1.02±0.13^{aB}	1.13±0.09^{aA}
	DPG2	0.07±0.01^{bD}	0.08±0.01^{bD}	0.11±0.01^{bC}	0.67±0.09^{bB}	0.85±0.04^{bA}
	LP100R	0.08±0.01^{bD}	0.09±0.01^{bD}	0.12±0.01^{bC}	0.16±0.01^{cB}	0.43±0.05^{cA}
n-Butyric acid	Blank	1.16±0.02^{aC}	1.57±0.08^{cA}	1.27±0.06^{eB}	1.26±0.04^{eB}	1.27±0.05^{eB}
	GOS	1.10±0.01^{bC}	2.78±0.12^{aB}	2.93±0.02^{aA}	2.77±0.11^{dB}	2.76±0.05^{dB}
	AEPG2	1.07±0.01^{cE}	1.18±0.11^{eD}	1.38±0.08^{dC}	5.54±0.66^{aB}	7.98±0.16^{aA}
	DPG2	1.04±0.03^{cE}	1.45±0.03^{dD}	1.59±0.08^{cC}	5.40±0.31^{bB}	7.05±0.30^{bA}
	LP100R	1.04±0.03^{cE}	2.00±0.17^{bD}	2.77±0.16^{bC}	3.41±0.09^{cB}	4.24±0.19^{cA}
i-Valeric acid	Blank	0.15±0.01^{aC}	0.15±0.01^{cC}	0.18±0.03^{dB}	0.23±0.03^{eA}	0.25±0.01^{eA}
	GOS	0.15±0.01^{aC}	0.46±0.02^{aA}	0.36±0.02^{aB}	0.32±0.03^{dB}	0.32±0.03^{dB}
	AEPG2	0.16±0.01^{aC}	0.12±0.01^{dC}	0.23±0.03^{cB}	1.53±0.17^{aA}	1.51±0.08^{aA}
	DPG2	0.16±0.01^{aD}	0.19±0.10^{bC}	0.20±0.02^{cC}	1.07±0.10^{bB}	1.23±0.08^{bA}
	LP100R	0.15±0.01^{aD}	0.18±0.02^{bD}	0.27±0.03^{bC}	0.41±0.03^{cB}	0.85±0.07^{cA}
n-Valeric acid	Blank	0.23±0.02^{bB}	0.14±0.01^{cD}	0.28±0.02^{bA}	0.26±0.02^{cA}	0.18±0.02^{dC}
	GOS	0.25±0.02^{aD}	0.59±0.01^{aB}	0.47±0.05^{aC}	0.48±0.04^{bC}	0.92±0.08^{aA}
	AEPG2	0.22±0.01^{bC}	0.17±0.01^{bD}	0.17±0.01^{cD}	0.52±0.07^{aB}	0.86±0.03^{bA}
	DPG2	0.22±0.01^{bC}	0.16±0.01^{bD}	0.17±0.01^{cD}	0.46±0.06^{bB}	0.94±0.10^{aA}
	LP100R	0.21±0.01^{bC}	0.17±0.01^{bD}	0.19±0.01^{cD}	0.28±0.03^{cB}	0.70±0.11^{cA}
Total SCFAs	Blank	7.73±0.19^{bD}	9.92±0.24^{cC}	10.71±0.11^{cB}	10.79±0.20^{eA}	10.80±0.30^{eA}
	GOS	7.72±0.11^{bE}	23.47±1.58^{aD}	30.04±0.82^{aC}	31.34±0.86^{cB}	33.68±0.47^{cA}
	AEPG2	7.81±0.02^{aE}	8.52±0.25^{eD}	9.42±0.16^{eC}	34.79±4.19^{bB}	52.23±0.91^{aA}
	DPG2	7.80±0.12^{aE}	9.64±0.22^{dD}	10.24±0.21^{dC}	39.25±1.63^{aB}	49.87±2.30^{bA}
	LP100R	7.72±0.14^{bE}	14.36±0.44^{bD}	18.95±0.70^{bC}	25.30±1.03^{dB}	31.50±1.07^{dA}

Different lowercase letters indicate significant differences ($p < 0.05$) among different groups. Different capital letters indicate significant differences ($p < 0.05$) in the same group among different time points. n=3.
